# Extending the IEEE 802.15.4 Security Suite with a Compact Implementation of the NIST P-192/B-163 Elliptic Curves

**DOI:** 10.3390/s130809704

**Published:** 2013-07-29

**Authors:** Antonio de la Piedra, An Braeken, Abdellah Touhafi

**Affiliations:** 1 Department of Electronics and Informatics (ETRO), Faculty of Engineering Sciences, Vrije Universiteit Brussel (VUB), Pleinlaan 2, Brussels 1050, Belgium; E-Mail: abdellah.touhafi@ehb.be; 2 Erasmushogeschool Brussel, Nijverheidskaai 170, Brussels 1070, Belgium; E-Mail: an.braeken@ehb.be

**Keywords:** wireless sensor networks, FPGA, 802.15.4

## Abstract

Typically, commercial sensor nodes are equipped with MCUsclocked at a low-frequency (*i.e.*, within the 4–12 MHz range). Consequently, executing cryptographic algorithms in those MCUs generally requires a huge amount of time. In this respect, the required energy consumption can be higher than using a separate accelerator based on a Field-programmable Gate Array (FPGA) that is switched on when needed. In this manuscript, we present the design of a cryptographic accelerator suitable for an FPGA-based sensor node and compliant with the IEEE802.15.4 standard. All the embedded resources of the target platform (Xilinx Artix-7) have been maximized in order to provide a cost-effective solution. Moreover, we have added key negotiation capabilities to the IEEE 802.15.4 security suite based on Elliptic Curve Cryptography (ECC;. Our results suggest that tailored accelerators based on FPGA can behave better in terms of energy than contemporary software solutions for *motes*, such as the TinyECC and NanoECC libraries. In this regard, a point multiplication (PM) can be performed between 8.58- and 15.4-times faster, 3.40- to 23.59-times faster (Elliptic Curve Diffie-Hellman, ECDH) and between 5.45- and 34.26-times faster (Elliptic Curve Integrated Encryption Scheme, ECIES). Moreover, the energy consumption was also improved with a factor of 8.96 (PM).

## 1. Introduction

Wireless Medical Sensor Networks (WMSNs) have several benefits. New medical infrastructure can replace wired telemetry applications. This is important in fields related to ambulatory monitoring or rehabilitation, where WMSNs can provide additional flexibility [1]. Moreover, the same technology can be used in several situations. That means that once an in-home network has been deployed, the same connectivity can be used for emergency situations and be adapted to monitor the patient's evolution. Consequently, the deployment of WMSNs alters the space-temporal dimensions of the traditional medical infrastructure. In this respect, the patients do not have to go regularly to the hospital, since the doctors can receive information about the patient without his/her physical presence. Moreover, homes are reshaped into monitoring centers. Further, WMSNs can be used for faster detection of diseases, as well as for detecting minimal changes in the parameters being monitored [2]. Furthermore, vulnerable patients, such as infants and senior citizens, can be monitored in order to detect falls via physical activity monitoring systems [3].

Generally, medical applications utilize commercial sensor nodes based on low-power MCUs. Further, these nodes generally utilize a 2.4 GHz transmitter based on the IEEE 802.15.4 communication protocol [4]. However, due to the low frequency of the MCUs utilized therein, several practitioners have proposed the utilization of Field-programmable Gate Arrays (FPGAs) in node construction for accelerating a myriad of algorithms, ranging from image processing techniques to cryptographic primitives [5]. These nodes can be either based on the combination of a low-power MCU and FPGAs, e.g., [6,7], or purely based upon FPGAs [8]. However, the former have several advantages over the latter, since the MCU can set the FPGA in suspend or sleep mode, while the accelerating operation is not required, thus saving power.

In this manuscript, we proposed investigating the role of FPGAs in the development of infrastructure for sensor networks. In this respect, we explore a variety of topics:

1. How an authentication-encryption (AE) mode of a block cipher (AES) can be implemented by maximizing the utilization of the embedded resources of the FPGA, such as the DSPblocks (Section 3).
2. How finite field arithmetic (e.g., addition and multiplication) can be implemented through the DSP blocks of the FPGA for achieving a reduction in area (Section 4).
3. How cryptographic accelerators can be implemented in FPGA-based nodes or nodes based on the combination of MCU and FPGA for extending the IEEE 802.15.4 security suite with key establishment schemes (Section 4.4).

Finally, we present the design of a cryptographic core, implemented in VHDLand utilizing the described components. All the resources of the FPGA are optimally used for the implementation of the different cryptographic algorithms, based on known designs, with a good trade-off between speed and area. The proposed design can be used to accelerate and perform massive encryption and authentication primitives in applications with a large number of nodes, such as a patient monitoring application, either based on a Wireless Sensor Network (WSN) or Wireless Body Area Network (WBAN).

This manuscript is structured as follows. First, in Section 2, we describe other implementations of the IEEE 802.15.4 security suite that have been proposed in the literature and summarize our contributions. Then, in Section 3, we outline our implementation. In Section 4, we detail the proposed implementation of the NIST P-192 and B-163 curves. Finally, in Section 5, we arrange the designs sketched out in Sections 3 and 4 together. This results in a cryptographic accelerator compliant with the IEEE 802.15.4 standard and extended with Elliptic Curve Cryptography (ECC) capabilities that can be compared with other implementations in the literature. Finally, we describe our future work in Section 6 and end in Section 7 with some conclusions.

## 2. Related Work

Several authors have proposed FPGA-based designs compliant with the IEEE 802.15.4 in the literature. Hamalainen *et al.* proposed an implementation of the IEEE 802.15.4 security relying on the Altera Cyclone I FPGA [9]. The authors utilized a folded implementation of the AES. Their implementation consumes 98.92 mW clocked at 50 MHz. On the other hand, Song *et al.* utilized the Altera Stratix I FPGA [10]. They also relied on the AES folded architecture. At 3 MHz, their design consumes 29 mW.

Our design attempts to improve those architectures according to the following facts. First, we have selected a low-power FPGA (Artix-7). In contrast, Song *et al.* and Hamalainen *et al.* utilized high-end and large FPGAs, which we believe are ill-suited for node construction. Second, we have maximized the utilization of the FPGA embedded resources, such as the DSP blocks and the BRAM. In so doing, the overall area of our design has been reduced, as depicted in Section 5. Third, the target FPGA provides a wide range of capabilities, such as different sleep modes (useful for a future MCU-FPGA node construction) together with partial reconfiguration (PR) support. In this respect, this could be used for altering the ECC parameters and adding new security primitives.

Besides, the utilization of DSPs for constructing large multipliers in cryptographic designs is not uncommon in the literature. Güneysu *et al.* leveraged the DSP48 slice of the Virtex FPGA for accelerating the arithmetic of the P-256 and P-224 NIST curves, achieving the maximum frequency supported by the platform (490 MHz) [11]. The design of their multiplier exploits the Multiply-and-accumulate (MACC) mode of the DSPs for generating all the partial products in parallel. We have followed this approach in our design of the P-192 accelerator (Section 4.2.3). However, we have deactivated the first pipeline stage of the DSP block for reducing the number of cycles required to perform a multiplication, since our design is expected to run at a low frequency. Finally, Moore *et al.* followed a similar approach in the Virtex-7 FPGA [12]. Besides, Dinechin *et al.* extended the utilization of DSP blocks for implementing large multipliers based on the Karatsuba algorithm [13]. However, as Güneysu *et al.* claimed, there is no point in trading multiplications for additions given the full capabilities of the DSP block for performing both operations at the same speed and resource cost.

## 3. The IEEE 802.15.4 Security Suite

The IEEE 802.15.4 standard utilizes cryptographic techniques based on symmetric-key cryptography for ensuring data confidentiality, authenticity, integrity and replay protection [4]. All the security suites utilize a symmetric block cipher mode based on the AES using 128-bit keys [14]. The AES is utilized for performing both encryption and authentication through the CCMmode [15]. This mode relies on the Counter (CTR) mode for ensuring confidentiality, whereas the Cipher Block Chaining (CBC) mode is utilized for generating an authentication tag.

### 3.1. AES

The AES-128 requires 10 rounds for each encryption process. In each round, four different operations manipulate an internal state of 16 bytes. These operations are based on the *GF*(2^8^) extension field. The elements of this field are expressed as polynomials according to the form *A*(*x*) = *a*_7_*x*^7^ + … + *a*_1_*x* + *a*_0_. The set of coefficients of each polynomial forms an eight-bit vector, represented in *GF*(2). Consequently, all the AES arithmetic is performed on both the *GF*(2^8^) and *GF*(2) fields. The internal state of the AES is represented by a 4 × 4 matrix, where each element forms an eight-bit vector.

Only the encryption part of the AES is reviewed here, since its decryption part is not utilized in the CCM mode. The inner four operations of each round in the AES encryption are the following. The *AddRoundKey* operation mixes the plain-text with the subkey, derived from the key schedule. Then, the *SubBytes* operation adds non-linearity to the block cipher by replacing each byte of the state with a unique element. This substitution is generally implemented using 256 × eight-bit substitution boxes. However, this substitution is based on two arithmetic operations. These operations encompass a *GF*(2^8^) inversion in tandem with an *affine* mapping. This *affine* mapping requires a *GF*(2^8^) multiplication and the addition of an eight-bit constant (*cf.*, [16]). Finally, the *ShiftRows* operation together with the *MixColumns* operation add diffusion to the AES internal state. The *ShiftRows* operation is based on a circular shift of the state, whereas the *MixColumns* operations modifies each four-byte column of the state via *GF*(2^8^) multiplications of a 4 × 4 matrix made of constants.

The *KeySchedule* operation generates 11 subkeys that are used in the ten rounds of AES-128. The generation is recursive, and each subkey is generated in four words of 32 bits. A function (namely g) adds non-linearity to the process using four substitution boxes from the *SubBytes* operation together with the addition of a variable coefficient (RCON). Finally, the generated subkeys are XORedwith the internal state in each round.

**Figure 1.**
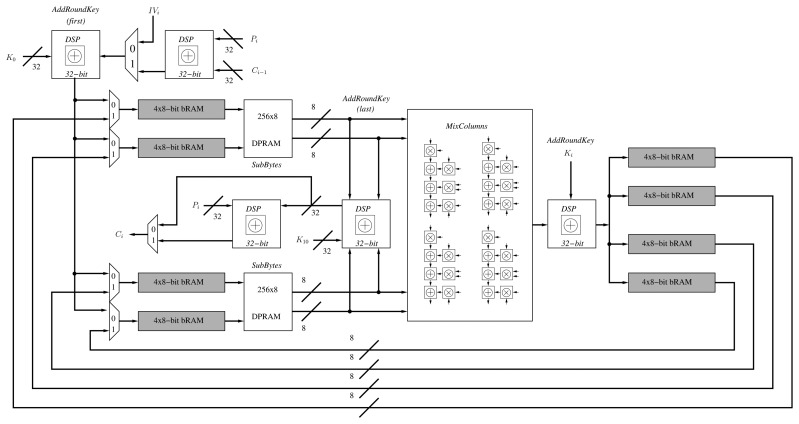
Organization of the proposed AES-CCM architecture.

By using the AES *folded* architecture, it is possible to reduce the implementation area by four. Generally, 16 S-BOXes are required to implement the *SubBytes* operation in one cycle. However, it is possible to implement only four substitution boxes and generate 32 bits of the state per cycle. Likewise, it is possible to reduce the number of *MixColumns* operations to only one. Moreover, the *AddRoundKey* operation is reduced from an XOR operation of 128 bits to a 32-bit XOR gate. Finally, the *ShiftRows* operation is performed by a special arrangement of the AES internal state at the beginning of each round. Hence, the encryption operation of a single block of 128 bits requires 60 cycles, i.e., 10 × 4 = 40, together with two extra cycles per round, due to the latencies of both substitution boxes and input/output memories of the folded register. Besides, we have optimized the AES data-path via DSP blocks in two ways. First, we have replaced the *AddRoundKey* operation by one DSP block in XOR mode. Second, we have extended the utilization of the DSP blocks to the computations of the *MixColumns* operation (Figure 1).

**Figure 2.**
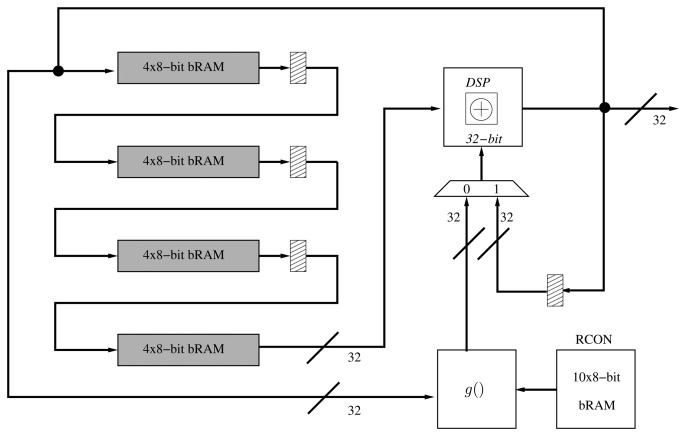
Proposed organization for the key schedule.

The architecture of the *KeySchedule* operation can also be implemented following an iterative approach by computing a quarter of the subkey in each clock cycle. This implementation, based on [17], computes 32 bits of key material per cycle, thus requiring 55 clock cycles to derive the complete set of subkeys ((4 + 1) × 11 = 55). This architecture requires a shift register that processes each 32-bit word before an XOR operation is performed. In order to reduce the area, we have implemented a shift-register totally based on BRAM (Figure 2). As in the folded register, we have replaced the 32-bit XOR operation of the key schedule with a DSP block.

Since the CCM mode only requires the encryption part of AES, it can be implemented with two extra XOR gates of 32 bits (Figure 1). One is for the CBC operation and the beginning of the encryption. The other XOR gate is placed at the end for the XORing operation with the output of AES-CTR. Finally, two multiplexers select the input/output of the AES encryption process according to the mode (CBC or CTR).

## 4. Implementation of Finite Field Arithmetic for ECC

In this section, we describe how the finite field arithmetic of two standardized curves (particularly the B-163 and the P-192 curves [18]) can be implemented mainly based on DSP blocks.

ECC was independently proposed by Victor Miller in 1985 and by Neal Koblitz in 1987 [19,20]. It provides the same level of security of RSAvia smaller key lengths and a reduced set of operations. Hence, the utilization of ECC in area- and power-constrained systems, such as RFIDand sensor nodes, is commonplace.

Elliptic Curves (ECs) are generally represented over prime fields (*i.e.*, *GF*(*p*) or (image)*_p_*, where *p* is prime) and binary extension fields (*GF*(*2^m^*) or (image)_2*^m^*_). The latter is generally preferred for hardware implementations, since the main operations are based on logic functions and shifts.

Prime fields in the form of *GF*(*p*) consist of a set of integers, 0, …, *p* – 1, where *p* is prime. Both the addition and multiplication operations are performed modulo *p*. For instance, all the operations in the in the P-192 curve are performed modulo *p*_192_ = 2^192^ − 2^64^ − 1 [18].

On the other hand, in binary extension fields in the form of *GF*(2*^m^*), the elements of the field are represented as polynomials, where modular reductions are replaced by a reduction through an irreducible polynomial. In the case of the B-163, with *m* = 163, the irreducible polynomial is represented as x^163^ + *x*^7^ + *x*^6^ + *x*^3^ + 1 [18].

However, in order to optimize the implementation of ECC arithmetics and avoid implementing the division operation, a number of inverse-free coordinate systems have been proposed in the literature. The importance of selecting a coordinate system stems from the fact that a reduced number of either additions or multiplications is preferred in an energy-constrained design. Therefore, in order to reduce the number of cycles required for performing a point operation in a cryptographic implementation, it is important to carefully choose the coordinate system. In the next section, we describe a number of coordinate systems generally utilized in the literature. We utilize [21] as a reference.

### 4.1. Selecting an Appropriate Coordinate System

Elliptic curves over prime fields, (*GF*(*p*)), are represented by the following equation:

(1) $$E:y^{2}=x^{3}+a_{4}\times x+a_{6}$$

where *p* is prime, *p* > 3 is satisfied and *a*_4_, *a*_6_ ∈ (image)*_p_*.

Standard projective coordinates utilize triples represented by (*x*_1_, *y*_1_, *z*_1_). They are derived from an *affine* point given by $\left(\frac{x_{1}}{z_{1}},\frac{y_{1}}{z_{1}}\right)$for *z*_1_ ≠ 0. In this system of coordinates, the number of operations for a point addition (PA) consists of 12 multiplications (M) and two squarings (S), whereas it requires seven multiplications (7M) and five squarings (5S) for performing a point doubling (PD). Besides, Jacobian coordinates utilizes triples, (*x*_1_, *y*_1_, *z*_1_), derived from the $\left(\frac{x_{1}}{z_{1}^{2}},\frac{y_{1}}{z_{1}^{3}}\right)$ *affine* point, where *z*_1_ ≠ 0. The PA and PD require 12M + 4S and 8M + 3S operations, respectively.

Finally, Chudnovsky-Jacobian coordinates utilize points represented with five coordinates *i.e.*, (*x*_1_, *y*_1_, *z*_1_, $z_{1}^{2}$, $z_{1}^{3}$). The PA operation is performed via 11M + 3S operations, whereas a PD is performed through 5M + 6S operations. Table 1 summarizes the number of operations of the coordinate systems described in this section.

On the other hand, in (image)_2_*_m_*, the following elliptic curve is generally utilized:

(2) $$E:y^{2}+x\times y=x^{3}+a_{2}\times x^{2}+a_{6}$$

where *a*_2_, *a*_6_ ∈ *F*_2_*_m_*.

Similarly to prime fields, projective coordinates and Jacobian ones can be utilized. Standard projective coordinates require 16M + 2S (PA) and 8M + 4S (PD) operations, whereas using the Jacobian system of coordinates, a PA is performed in 16M + 3S operations and 11M + 3S, in the case of PD. Besides, the López-Dahab (LD) system of coordinates derives the triple, (*x*_1_, *y*_1_, *z*_1_), from the *affine* point, $\left(\frac{x_{1}}{z_{1}},\frac{y_{1}}{z_{1}^{2}}\right)$, where *z*_1_ ≠ 0. Performing a PA via LD coordinates requires 13M + 4S operations, whereas PD is performed in 5M + 4S operations. Table 2 summarizes the number of operations of the coordinate systems described in this section.

According to Table 1 and Table 2, we have selected a pair of systems of coordinates suitable for the implementation of the P-192 and the B-163 curves. In the case of the P-192 curve, we have chosen projective coordinates. The Jacobian system of coordinates requires a large number of operations, whereas the Chudnovsky-Jacobian, despite the reduction in the number of multiplications, requires five points per coordinate, which greatly increases the area of the implementation for storing them.

In the case of the B-163 curve, we have selected the LD coordinates, since it requires a reduced number of multiplications in comparison with the standard projective and Jacobian coordinates (Table 2).

### 4.2. Design of Finite Field Arithmetic over *GF*(*p*192)

In this section, we describe our implementation of the P-192 curve operations. These components are utilized for extending the IEEE 802.15.4 security suite using key negotiation schemes based on ECC.

#### 4.2.1. Modular Addition and Subtraction

Integer modular addition and subtraction are performed mod *p*_192_ = 2^192^ −2^64^ −1 in the P-192 curve. Algorithms 1 and 2 represents both modular addition and subtraction mod *p*_192_.



**Algorithm 1** Integer modular addition.
**Input:** Integers (a, b), represented as binary vectors in the form *a* = (*a*_191_, …, *a*_0_) and *b* = (*b*_191_, …, *b*_0_), modulus p_192_ = 2^192^ − 2^64^ − 1.**Output:***c* = *a* + *b* mod *p*_192_.1:*c*_1_ = *a*+*b*2:*c*_2_ = *c*_1_ − *p*_192_3:**if***c*_2_ ≥ 0 **then**4: **return***c*_2_5:**else**6: **return***c*_1_7:**end if**


**Algorithm 2** Integer modular subtraction.
**Input:** Integers (*a*, *b*), represented as binary vectors in the form *a* = (*a*_191_, …, *a*_0_) and *b* = (*b*_191_, …, *b*_0_), modulus *p*_192_ = 2^192^ − 2^64^ − 1.**Output:**
*c* = *a* − *b* mod *p*_192_.1:*c*_1_ = *a* − *b*2:*c*_2_ = *c*_1_ + *p*_192_3:**if***c*_1_ < 0 **then**4: **return***c*_2_5:**else**6: **return***c*_1_7:**end if**


The DSP48E1 block [22] allows us to perform 48-bit additions and subtractions with carry input and output. If we cascade several DSP blocks and connect the carry input and output signals, it is possible to construct larger multipliers. Consequently, given the carry support in the block, we do not need to implement additional logic for accelerating its computation. This is the case of the Carry-Lookahead Adder (CLA) and conditional sum adders that implement extra logic for accelerating the computation of the carry [23]. Moreover, high-speed architectures, such as prefix adders (e.g., Brent-Kung and Kogge-Stone), are based on binary logic bit-wise operations that undermine the utilization of the DSP blocks for implementing them [23].

**Figure 3.**
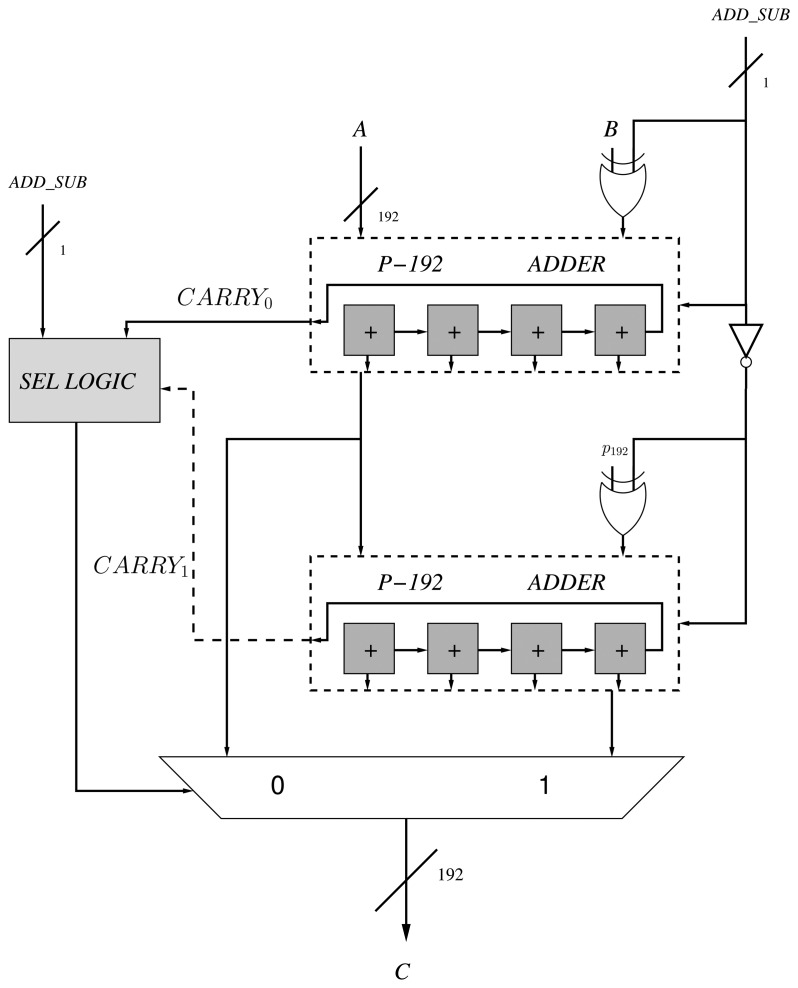
*p*_192_ modular adder and subtractor [24].

Consequently, we utilize the DSP blocks as 48-bit with carry support. We have utilized four DSP blocks for implementing a full operation of 192-bit. In order to optimize the design of the adder/subtractor and perform both operations using only one component, we rely on the design proposed by [24]. This design requires two adders (instead of one adder and one subtractor or a configurable adder) of *k* length and a combinational circuit that selects the output according to the addition or subtraction operation. The authors have replaced the second operand, *b*, by 2*^k^* − *b* − 1 in the subtraction operation, and *c*_2_ is computed as *c*_1_ + (2*^k^* −*p*_192_) instead of *c*_1_ −*p*_192_ in the addition process (Figure 3).

The addition of two operands (e.g., A and B) requires one cycle in the DSP block. Then, an extra cycle is required to propagate the carry among the blocks. Consequently, four cycles are required for performing one modular addition or subtraction, since there are two 192-bit adders in the proposed design.

#### 4.2.2. Modular Reduction

The NIST curves utilize pseudo-Mersenne primes for performing fast reductions using only additions and subtractions [18]. The NIST algorithm for performing reductions in the P-192 curve is depicted in Algorithm 3.

The reduction consists of four additions that can be executed in the adder/subtractor. Consequently, a modular reduction can be achieved in 16 cycles.



**Algorithm 3** Modular reduction *p*_192_.
**Input:** An integer represented as *a* = (*a*_0_, …, *a*_6_), where *a_i_* has a length of 64-bit.**Output:***a* mod *p*_192_1:*c*_0_ = (*c*_2_,*c*_1_, *c*_0_)2:*c*_1_ = (0, *c*_3_, *c*_3_)3:*c*_2_ = (*c*_4_, *c*_4_, 0)4:*c*_3_ = (*c*_5_,*c*_5_, *c*_5_)5:**return***c*_0_ + *c*_1_ + *c*_2_ + *c*_3_ mod *p*_192_


#### 4.2.3. Modular Multiplication Operation

The DSP48E1 block supports 25 × 18-bit multiplications, which can optionally be coupled with a 48-bit accumulator. Generally, the multiplication operation is based on two main operations. First, a group of partial products are computed. Then, they are shifted and accumulated for generating the final result.

In the literature, multiplication techniques are generally categorized among parallel and sequential multipliers [23]. Sequential multipliers process one bit at a time of one of the operands in each cycle, *i.e.*, this bit is multiplied by the second operand, shifted and accumulated. Other algorithms, such as the Booth's multiplier, process two bits per cycle by applying a transformation to certain bit patterns in the operands [25]. Moreover, other variants, such as the radix-4 and radix-8 Booth's multipliers, extend the number of bits being processed at a time [26]. However, since we can compute the complete multiplication of two operands of 18-bit in one cycle, implementing any sequential multiplication algorithm would not take advantage of the full features of the DSP block. On the other hand, parallel multipliers generate all the partial products in parallel and accumulate them.

Given that we can process a 25 × 18-bit product at a time, we can use several DSPs for generating and accumulating the partial products in parallel. In this case, since we work with 192-bit operands, they can be decomposed in 16 segments of 16-bit and be processed using 16 × 16-bit multiplications. This decomposition is based on the addition of 12 segments shifted *k* bits, according to their position in the operand:

(3) $$A=2^{11k}\times a_{11}+\ldots +2^{k}\times a_{1}+a_{0}$$

(4) $$B=2^{11k}\times b_{11}+\ldots +2^{k}\times b_{1}+b_{0}$$

If we operate the product, *A* × *B*, we obtain 12^2^ = 144 partial products that can be added according to the displacement, 2*^k^*, ranging from 2*^k^* to 2^22^*^k^*. Consequently, we require 23 DSP blocks in Multiply-and-accumulate (MACC) mode for generating all the partial products in parallel, e.g.:

(5) $$\text{MAC}C_{0}=a_{0}\times b_{0}$$

(6) $$\text{MAC}C_{1}=2^{k}\times a_{1}\times b_{0}+2^{k}\times a_{0}\times b_{1}$$

(7) $$\text{MAC}C_{2}=2^{2k}\times a_{2}\times b_{0}+2^{2k}\times a_{1}\times b_{1}+2^{2k}\times a_{0}\times b_{2}$$

(8) …

Finally, 23 accumulated partial products can be added together for obtaining the final result. This is done using one DSP block in addition mode. This operation is based on shifting each partial product *2^ik^* bits for *k* = 16, e.g., *A* × *B* = (*MACC*_23_*_k_* ≪ 23*k*) + … + (*MACC*_1_ ≪ *k*) + *MACC*_0_.

**Figure 4.**
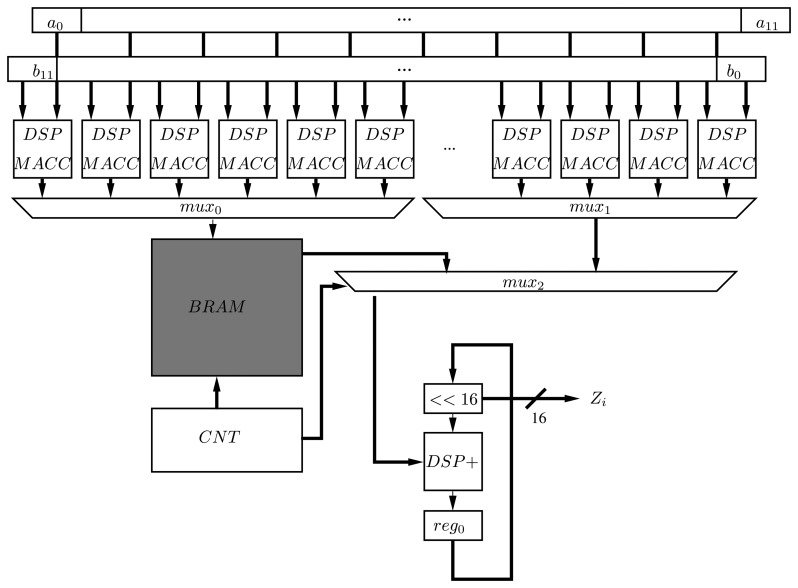
One-hundred and ninety-two-bit multiplier design.

Each MACC operation requires an initial delay (one cycle) to fill the pipeline of the DSP block and an extra cycle for each subsequent multiplication and addition. At the same time, the results of each MACC are accumulated in another DSP block, selected by a multiplexer coupled to a counter. However, given that the first half of partial accumulations (*MACC*_0–11_) and the second one (*MACC*_12–22_) are being generated at the same time, the second part is stored, while the first one is processed in a BRAM. Then, this BRAM is read through a counter and added (Figure 4). Finally, two shift registers are utilized to route the 16-bit segments of each operand (*A*, *B*) to the MACCs.

Since all the partial products are computed in parallel and are being added after the first partial product is generated (*M*_0_), the number of cycles for computing a multiplication is 23+1 (delay MACC) +1 (DSP addition) = 25 cycles.

### 4.3. Design of Finite Field Arithmetic over *GF*(2^163^)

In this section, we describe how we have implemented the different units for performing operations in *GF*(2^163^). These operations are then contrasted with those of the NanoECC and TinyECC libraries in Section 5.

#### 4.3.1. Addition

**Figure 5.**
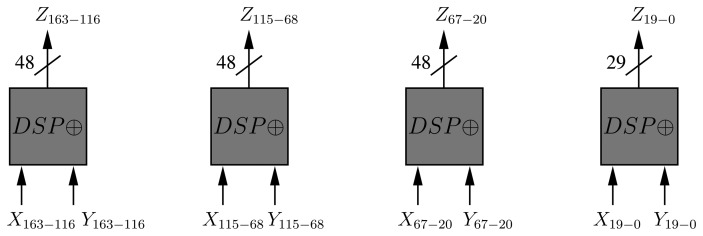
Organization of the proposed B-163 adder.

Addition in *GF*(2*^m^*) is simply performed via an XOR operation (Algorithm 4). Given that one DSP block can process up to 48 bits, four blocks in XOR mode are enough for implementing an addition operation in *GF*(2^163^) (Figure 5). This operation requires one cycle.



**Algorithm 4***GF*(2*^m^*) addition.
**Input:***X*, *Y*, *Z* ∈ *GF*(2^163^).**Output:***Z* = *X* + *Y*.1:*Z* ← *X* ⊕ *Y*2:**return***Z*


**Algorithm 5** Bit-serial *GF*(2^163^) multiplication ∀*A*, *B*, *C*, *M* ∈ *GF*(*2*^163^), *M* = *x*^163^ + *x*^7^ + *x*^6^ + *x*^3^ + 1.
**Input:** Two 163-bit vectors *A* = (*a*_0_, …, *a*_162_), *B* = (*b*_0_, …, *b*_162_) ∈ *GF*(2^128^).**Output:** One 163-bit vector *C* = (*c*_0_, …, *c*_162_) ∈ *GF*(2^163^).1:*C* ← 02:**for***i* = 0 → 162 **do**3: **if***B_i_* = 1 **then**4:  *C* = *C* ⊕ *A*5: **end if**6: **if***A*_162_ = 1 **then**7:   (*A* ≪ 1) ⊕ *M*8: **else**9:  *A* ≪ 110: **end if**11:**end for**12:**return***C*


#### 4.3.2. Multiplication

In our design, the *GF*(2^163^) multiplication operation is performed using a bit-serial approach. The product is then reduced by an irreducible polynomial (*g*(*x*) = *x*^163^ + *x*^7^ + *x*^6^ + *x*^3^ + 1, *cf.*, [18]). The proposed multiplier utilizes eight DSP blocks for performing the required 163-bit XOR operations. Since we use a bit-serial approach, our design requires 163 cycles for performing a multiplication, according to Algorithm 5.

### 4.4. Proposed EC Schemes

The IEEE 802.15.4 standard does not describe how keys are generated. Those operations are supposed to be provided by the protocol upper layers. Since shared keys need to be renegotiated by the intended parties before the message counter overflows (*i.e.*, for ensuring key freshness), an efficient key agreement protocol must be implemented. In the proposed design, the ECDH, ECIES and Elliptic Curve Menezes-Qu-Vanstone (ECMQV) schemes can be implemented. We describe ECDH and ECIES, since they have already been implemented in commercial sensor nodes, and their capabilities are compared in Section 5.

#### 4.4.1. ECDH

ECDH is a key agreement protocol that establishes a shared secret between two non-authenticated parties. It follows a similar approach as the Diffie-Hellman (DH) key exchange [27]. In ECDH, each party randomly selects a secret value (*x* and *y*, respectively). Then, they compute *xG* and *yG* given *G* as the primitive element of the curve (This element is the generator of the multiplicative group of the finite field.). Both values, *xG* and *yG*, are exchanged, and a shared secret, *k*, is computed as *x*(*yG*) and *y*(*xG*) due to the associative property of the point multiplication. Both *x* and *y* values are considered private keys.

The strength of ECDH resides in the Elliptic Curve Discrete Logarithm Problem (ECDLP), *i.e.*, finding an integer, *z*, where *zG* = *C* and *C* is another element of the field by computing the discrete logarithm, *z* = *log_G_*(*zG*) (A summary of several methods for solving discrete logarithms can be found in [28].).

#### 4.4.2. ECIES

On the other hand, ECIES is an authenticated encryption protocol based on EC. ECIES has been standardized by several organizations, such as ANSI, IEEE, SECG and ISO/IEC [29]. In this manuscript, we have selected the standard described by the Standards for Efficient Cryptography Group (SECG) [30]. This standard supports the same curves defined by the NIST, *i.e.*, both the B-163 and P-192 curves are therefore covered.

ECIES consists of three main components:

1. A primitive that generates a MACfor authenticating each message. It can be based on HMAC-secure hash algorithm (SHA)-1, HMAC-SHA-2 or AES-CBC-MAC. Since we have already implemented AES-CBC-MAC for supporting the IEEE 802.15.4 security suite, we have selected this technique.
2. A Key Derivation Function (KDF) that generates a shared key. In this case, two standards are supported: X.9.63-KDF and NIST 800-5. We have selected X.9.63-KDF, which consists of a message digest generation via SHA-1 or SHA-2. In this respect, we have implemented SHA-256 for computing the KDF (Section 4.4.3).
3. A symmetric encryption algorithm, either based on XOR or AES with 128, 192 and 256 key-lengths in CBC or CTR modes. Moreover, Triple DES (3DES) in CBC mode is also supported. Hence, we rely on AES-128 in CTR or CBC mode since it is available from the implementation of the IEEE 802.15.4 security suite (Section 3.1).

Moreover, two parameters are required before a message is sent from A to B:

1. The public key of party B generated as *K_B_* = *k_b_G*, where *k_b_* is considered the private key of B and *G* the field generator.
2. Additional information, represented as *S*_{1,2}_.

The first part of the scheme derives a shared secret, *S*, based on ECDH, which is used for generating and exchange two keys. The first key (*k_E_*) is utilized for encrypting a message, *m*, using a symmetric algorithm (via AES-CTR or AES-CBC). The second one (*k_MAC_*) is utilized for generating an authentication field. Both keys are derived as *k_E_*|*k_MAC_* = *KDF*(*S*║*S*_1_). Then, the message is encrypted and authenticated. Finally, the material for deriving the shared secret, *S*, is sent, concatenated with the encrypted message and the corresponding authentication code. Both the encryption and decryption processes are depicted in Algorithms 6 and 7.



**Algorithm 6** Elliptic Curve Integrated Encryption Scheme (ECIES) encryption operation (A).
**Input:** A random number, *r*, the public key of B (*K_B_*) and the generator of the field, *G*.**Output:** The material required for deriving the shared secret, *S*, together with the encrypted message, *c*, and the corresponding authentication code, *d*.1:*R* = *rG*2:*P* = (*P_x_*, *P_y_*) = *rK_B_*3:*S* =*P_x_*4:*k_E_*∣*k_MAC_* = *KDF*(*S*║*S*_1_)5:*c* = *AES*_k_(*k_e_*, *m*)6:*d* = *MAC_kMAC_* (*c*║*S*_2_)7:**return***R*║*c*║*d*


Consequently, the number of operations by a node that encrypts and sends a message through ECIES (A) consists of two point multiplications, the generation of keys (*k_E_*, *k_MAC_*) through SHA-256, the encryption of the message via AES-CTR and the generation of the authentication code using AES-CBC.



**Algorithm 7** ECIES decryption operation (B).
**Input:** The required material for deriving the shared secret, *S*, together with the encrypted message and its authentication code, (*R*║*c*║*d*).**Output:** The authenticated and original message, *m*.1:P = (*P_x_*, *P_y_*) = *Rk_b_* = *rGk_b_* = *rK_B_*2:*S* = *P_x_*3:*k_E_*∣*k_MAC_*= *KDF*(*S*║*S*_1_)4:**if***d* = *MAC_kMAC_*(*c*║*S*_2_) **then**5: *m* = *AES_k_*(*k_e_*, *c*)6: **return**
*m*7:**end if**8:**return**
*null*


#### 4.4.3. Message Digest Generation

**Figure 6.**
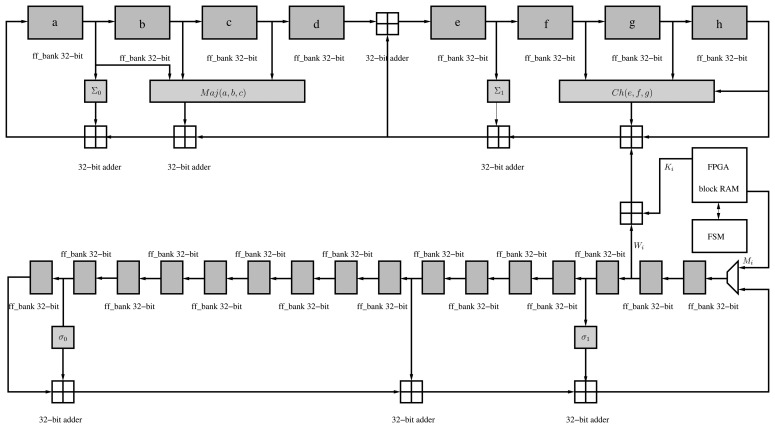
Organization of the secure hash algorithm (SHA)-256 implementation.

As noted before, SHA-256 has been implemented in the proposed accelerator to perform the KDF during the key establishment process. The secure hash algorithm, SHA-256, is part of the SHA-2 family, standardized by NIST [18]. A hash algorithm provides a fixed-length and unique representation of a message. This is also called a digest. SHA-256 processes blocks of 512 bits and generates a unique digest of 256 bits. The hash function consists of padding of the message in blocks of 512 bits and generating the message digest during 64 iterations. A predefined 32-bit constant (K*_i_*) is applied in each iteration in the main pipeline. Moreover, a message scheduler generates a 32-bit word, *W_j_*, in each iteration, which is then applied in order to generate the hash (Figure 6).

The message scheduler is initialized with the padded message at the beginning of the hash computation, whereas the main pipeline registers (**a**–**h**) are initialized with eight predefined words of 32 bits defined by the standard. The hash function involves the use of six logic functions (Ch, Maj, Σ_0_, Σ_1_, *σ*_0_ and *σ*_1_) defined in [18].

## 5. Results

**Figure 7.**
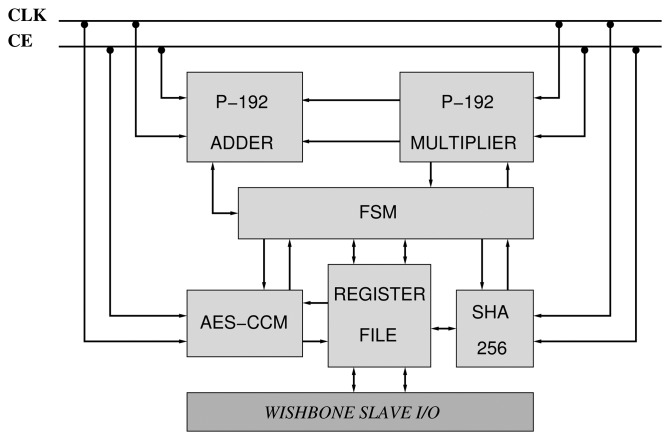
Organization of the P-192 accelerator.

We have constructed two accelerators based on the NIST curves, B-163 and P-192 (Figure 7). They are compliant with the IEEE 802.15.4 security suite. Consequently, the AES-CCM mode has been implemented according to the design presented in Section 3.1. Moreover, the designs of the arithmetics described in Sections 4.2 and 4.3 for *GF*(192) and *GF*(2^163^) have also been utilized. Finally, a Finite State Machine (FSM) orchestrates the execution of PA, PD and PM primitives between the different components of the core (Figure 7).

Since the number of pins available in the target FPGA (Artix-7) is not enough for supporting two input operands and one output operand of 128/163/192 bits, we rely on a simplified slave bus interface based on the Wishbone interconnection standard [31].

### 5.1. Software Power Analysis in Xilinx Platforms

We have performed software power analysis in the designs described in this manuscript through the Xilinx Power Analyzer (XPA) [32]. Given that dynamic power is not a stable value, the user must provide a simulation file (VCD) containing the value for the signals over an interval of time. We have obtained our VCD files through the Mentor ModelSim simulator. However, a VCD file derived from a standard simulation does not contain all the internal connections and elements that are mapped during the Place and Route (PAR) phase. Hence, it is mandatory to generate a post-PAR simulation model for each operation performed in the core for increasing the accuracy of the power figures.

### 5.2. PAR Results of the P-192 and B-163 Operations

We have depicted the PAR results of each implemented arithmetic circuit for performing operations on the P-192 and B-163 curves in Tables4. In this respect, Table 3 depicts the area figures for the circuits implemented only using LUTs. We have also depicted the number of BRAMs that we have utilized. In this respect, we have stored both the *p*_192_ modulus in three blocks of BRAM in the P-192 adder/subtractor. Moreover, the P-192 multiplier utilizes one block of BRAM for storing the second half of the partial of products, while the first part is being accumulated. Finally, the B-163 multiplier stores the *GF*(2^163^) irreducible polynomial in two blocks of BRAMs.

According to Table 4, we obtained different reductions in area, ranging from 56.08% in the P-192 multiplier to 13.14% in the B-163 multiplier. The reduction achieved in the P-192 multiplier is based on the amount of FPGA resources that the MACCs based on LUT require. Moreover, this suggests that larger reductions in area can be achieved, implementing larger multipliers together with B-163 adders. However, we must take into account that the P-192 multiplier relies on two shift registers for the input operands, which can affect the area requirements. Besides, by using larger operands, a larger register file in the bus slave interface is required. However, if there are available BRAMs, they can be used for both implementing the shift registers (as we do in the AES key schedule, Section 3.1) and the register file.

### 5.3. Area

Table 5 depicts the area figures of the P-192 and B-163 accelerators. Due to the extra logic that the P-192 arithmetic requires, e.g., two shift-registers in the case of the multiplier, the P-192 needs an additional amount of slices. Moreover, in the case of the B-163 adder, it is only implemented via DSP blocks, since the addition in *GF*(2*^m^*) is performed through XOR operations.

Finally, the area is also dominated by the slices required by the SHA-256 implementation together with the set of registers that stores three pairs of coordinates in projective and LD form. We have implemented all the 32-bit arithmetic and logic operations of the SHA-256 algorithm via XOR gates, obtaining a reduction in area of 19.91% (Table 6).

### 5.4. Power and Performance

We have generated a post-PAR simulation model of the P-192 and B-163 accelerators. First, we have simulated the execution of several operations for generating the corresponding signal activity file at 10 MHz. The selection of this frequency stems from the fact that this accelerator will run at the typical frequency that *motes* do [33]. Second, the VCD file has been fed into XPA for extracting the required power during the execution of each operation. The execution time for each operation includes the writing of the operands (coordinates) into the register file.

Table 7 and Table 8 depict the power consumption and energy per operation in both accelerators. The performance of the PM operation was measured using the *double-and-add* algorithm (Algorithm 8). We have depicted an average number of PD and PA operations, *i.e*., *t* PDs and 0.5*t* PAs.

Given the area utilization of the SHA-256 implementation, this is the component of the accelerator that requires more power (53 mW in the P-192 accelerator and 49 mW in the B-163). The rest of the operations are executed in the B-163 accelerator with a reduction of 2–8 mW in comparison with the P-192 implementation, according to the achieved reduction in area (Section 5.3). Moreover, despite that the B-163 operations are performed through smaller operands, the fact that the *GF*(2^163^) multiplication requires 19.25 *μ*s per operation undermines an improvement in the energy consumption in the case of the PM, ECDH and ECIES operations (which require three-times more energy in the B-163 accelerator). Nonetheless, the utilization of a parallel or hybrid multiplier for performing the *GF*(2^163^) multiplications can improve both the time and energy consumption.



**Algorithm 8** Double-and-add algorithm for point multiplication.
**Input:** An integer, *k*, of length *n* and point *P* ∈ *GF*(*p*) or *GF*(2*^m^*).**Output:** A point, Z = *kP* ∈ (*GF*(*p*) or *GF*(2*^m^*).1:*Z* ← *P*2:**for***i* = 0 → *n* − 1 **do**3: *Z* =*Z* + *Z*4: **if***k_i_* = 1 **then**5:  *Z* = *Z* + *P*6: **end if**7:**end for**8:**return**
*Z*


Finally, Table 9 and Table 10 depict a comparison of the main operations (PM, ECDH, ECIES and AES-128 encryption) between the proposed design and software implementations tested by [34–36].

As depicted in Table 9, the operations executed in our implementation are between 8.58- and 15.4-times faster (PM), 3.40- to 23.59-times faster (ECDH), 5.45- and 34.26-times faster (ECIES) and between 64.60- and 404-times faster in the case of AES. Furthermore, a considerable reduction in energy consumption (Table 10) is also shown.

Finally, it is worth noting that we are using the XC7A100TL FPGA, which is one of the largest platforms of the Artix-7 series. Rather, using the XC7A20S (2,500 slices, 60 DSP48E1) renders the selected platform ill-suited, since a better power consumption and price are expected. Nevertheless, this platform was not available at the time of writing.

## 6. Future Work

The utilization of FPGAs for sensor node construction adopts the typical threat model of FPGA-based systems. That means that an attacker generally can have two main interests in the platform: recovering the secret keys and disrupting the system. Consequently, the unused I/O pins of the FPGA must be protected against leakage, and they must reject any request. Moreover, the programming interface of the FPGA must be locked for non-authorized readings and updates. In this respect, since we are using an SRAMFPGA, an external non-volatile memory is required to store the FPGA configuration, and bitstream encryption must be activated to avoid tampering. Finally, anti-fuse and FLASH-based FPGAs can be used to avoid this problem, as well as to mitigate the impact of side-channel attacks. Moreover, a number of authors have proposed different techniques to avoid these attacks on FPGAs based on masking, hiding and utilizing random-based arithmetics [37–39]. Another issue not discussed here has to do with the generation of keys through random data. In this respect, a number of authors have proposed several designs. First, Pseudo-Random Number Generators (PRNGs), based on Linear Feedback Shift Registers (LFSRs), can be used if the seed's entropy is large enough. For instance, seed extraction from different natural phenomena has been proposed, such as nuclear decay or thermal noise [40]. FPGA-based designs of LFSRs are numerous in the literature; see, for instance, [41–43]. Second, True Random Number Generators (TRNGs) utilize a physical process for generating random data. Particularly, those based on FPGA focus on exploiting the imperfections of components and logic implementations, such as the jitter of PLLsand ring oscillators [44–48]. Finally, TRNG designs based on Physical Unclonable Functions (PUFs) have been also proposed, as well as those based on writing collisions in BRAMs [49–51].

## 7. Conclusions

In this manuscript, we have presented the design of two cryptographic accelerators suitable for FPGA-based nodes, extended with key negotiation capabilities. The proposed platform is based on the low-power Xilinx Artix-7 FPGA. Moreover, we have taken advantage of the DSP48E1 slice for reducing the area figures of our design. In this respect, we have replaced the logic functions in the AES folded architecture described by Chodowiec *et al.* [17], compacting even more the implementation of the encryption operation. Besides, a similar approach was followed for implementing the arithmetic of the NIST P-192 and B-163 curves. Finally, by clocking the FPGA at 10 MHz, the required energy for performing a number of cryptographic operations was smaller in comparison to several software alternatives for *motes*, such as the NanoECC and TinyECC libraries.

## Figures and Tables

**Table 1.** Performance of coordinate systems in prime fields. PA, point addition; PD, point doubling; M, multiplication; S, squaring.

**System**	**PA**	**PD**
Standard projective	12M + 2S	7M + 5S
Jacobian	12M+4S	8M+3S
Chudnovsky-Jacobian	11M + 3S	5M + 6S

**Table 2.** Performance of coordinate systems in binary extension fields.

**System**	**PA**	**PD**
Standard projective	16M + 2S	8M + 4S
Jacobian	16M+3S	11M+3S
López-Dahab	13M+4S	5M+4S

**Table 3.** Place and Route (PAR) results of the cryptographic algorithms implemented only using LUTs (XC7A100TL).

**Algorithm**	***f*_max_ (MHz)**	**Cycles**	**Area (Slices)**	**BRAMs**	**DSPs**
P-192 modular adder/subtractor	173.361	4	399	3	-
P-192 multiplier	188.460	25	986	1	-
B-163 adder/subtractor	410.231	1	219	-	-
B-163 multiplier	445.177	163	312	2	-

**Table 4.** PAR results of the cryptographic algorithms implemented only using DSPs (XCTA100TL).

**Algorithm**	*f_max_***(Mhz)**	**Cycles**	**Area (Slices)**	**Area reduction (%)**	**BRAMs**	**DSPs**
P-192 modular adder/subtractor	92.237	4	302	24.31	3	8
P-192 multiplier	188.460	25	433	56.08	1	24
B-163 adder/subtractor	224.298	1	132	39.72	-	4
B-163 multiplier	259.700	163	271	13.14	2	8

**Table 5.** PAR results of the two proposed accelerators.

	**P-192**	**B-163**
Platform	Artix-7 (XC7A100TL)	Artix-7 (XC7A100TL)
*f_max_* (MHz)	51.244	51.244
# of Slices	1,418	603
# of BRAMs (36 kb)	4	2
# of BRAMs (18 kb)	20	21
# of DSP48A1 slices	63	38

**Table 6.** PAR results of the SHA-256 implementation.

	**LUT**	**DSP**
Platform	Artix-7 (XC7A100TL)	Artix-7 (XC7A100TL)
*f_max_* (MHz)	96.834	42.817
# of Slices	688	551
# of BRAMs (36 kb)	-	-
# of BRAMs (18 kb)	9	9
# of DSP48A1 slices	0	32

**Table 7.** Performance summary of the P-192 accelerator at 10 MHz. ECDH, Elliptic Curve Diffie-Hellman.

**Operation**	**Time (us)**	**Power (dynamic/total) (mW)**	**Energy (mJ)**
AES	5.55	8/45	2.49 × 10^-4^
SHA-256	9.45	15/53	5 × 10^-4^
Multiplication	4.65	10/47	2.18 × 10^-4^
Addition	2.85	7/47	1.33 × 10^-4^
Point addition	72.25	8/46	0.003
Point doubling	86.75	10/48	0.004
Point multiplication	23,056	10/48	1.10
ECDH	45,112	10/48	2.21
ECIES	46,129	10/48	2.21

**Table 8.** Performance summary of the B-163 accelerator at 10 MHz.

**Operation**	**Time (us)**	**Power (dynamic/total) (mW)**	**Energy (mJ)**
AES	5.55	5/43	2.38 × 10^-4^
SHA-256	9.45	12/49	4.63 × 10^-4^
Multiplication	19.25	3/40	7.70 × 10^-4^
Addition	1.95	4/41	7.99 × 10^-5^
Point addition	252.95	2/40	0.01
Point doubling	319.55	2/40	0.01
Point multiplication	83,850.35	2/40	3.35
ECDH	167,700	2/40	6.70
ECIES	167,720	2/40	6.70

**Table 9.** Comparison on execution time (ms) with other Elliptic Curve Cryptography (ECC) and AES-128 implementations in commercial sensor nodes (B-163).

**Implementation**	**Point multiplication (ms)**	**ECDH (ms)**	**ECIES (ms)**		**AES-128 (ms)**	
**This work (163-bit)@10 MHz**	**83.85**	**167.70**		**167.72**	**0.005**
NanoECC (160-bit)-MICA2 [34]	1,270	-		-	-
NanoECC (160-bit)-Tmote Sky [34]	720	-		-	-
TinyECC (160-bit)-MICAz [35]	-	3,956.17		5,746.2	-
TinyECC (160-bit)-Tmote Sky [35]	-	2,075.5		3,590.42	-
TinyECC (160-bit)-Imote2 (13 MHz) [35]	-	571.28		915.31	-
Healy *et al.*-CC2420 [36]	-	-		-	0.32383
Healy *et al.*-MICAz [36]	-	-		-	2.022

**Table 10.** Comparison on energy consumption (mJ) with other ECC and AES-128 implementations in commercial sensor nodes (B-163).

**Implementation**	**Point Multiplication (mJ)**	**ECDH (mJ)**	**ECIES (mJ)**	**AES-128 (mJ)**
**This work (163-bit)@10 MHz**	**3.35**	**6.70**	**6.70**	**2.38 × 10^-4^**
NanoECC (160-bit)-MICA2 [34]	30.02	-	-	-
NanoECC (160-bit)-Tmote Sky [34]	7.95	-	-	-
TinyECC (160-bit)-MICAz [35]	-	94.95	137.91	-
TinyECC (160-bit)-Tmote Sky [35]	-	16.61	24.78	-
TinyECC (160-bit)-Imote2 (13 MHz) [35]	-	16.83	26.95	-
Healy et al.-CC2420 [36]	-	-	-	0.0084
Healy et al.-MICAz [36]	-	-	-	0.0525
